# Perceived Stress and Depressive Symptoms in Urban Chinese Women: The Buffering and Exacerbating Roles of Marital Quality

**DOI:** 10.3390/healthcare14152246

**Published:** 2026-07-23

**Authors:** Huimin Zhang, Li Zheng, Xiaohe Xu, Shujie Han

**Affiliations:** 1 Department of Sociology and Psychology, Sichuan University, Chengdu 610065, China; huimin.zhang@ufl.edu; 2 Department of Sociology and Demography, The University of Texas at San Antonio, San Antonio, TX 78249, USA; xiaohe.xu@utsa.edu (X.X.); shujie.han@my.utsa.edu (S.H.)

**Keywords:** depressive symptoms, marital quality, perceived stress, stress-buffering hypothesis, stress-exacerbating hypothesis, stress process model, urban Chinese women

## Abstract

**Highlights:**

**What are the main findings?**

- Positive marital interaction buffered stress-related depressive symptoms.
- Marital disagreement intensified the mental health effects of stress.

**What are the implications of the main findings?**

- Marital quality has both protective and harmful mental health effects.
- Family-based interventions may improve women’s psychological well-being.

**Abstract:**

**Background/Objectives**: This study examines the moderating effect of marital quality on the association between perceived stress and depressive symptoms among married women residing in urban China, guided by the stress process model. **Methods**: Using data from the 2020 Family and Health Survey conducted in Chengdu and Beijing, the current study analyzed responses from 1865 married women. Marital quality was measured using two dimensions: positive marital interaction and marital disagreement. Ordinary Least Squares regression analyses showed that higher perceived stress was significantly associated with elevated depressive symptoms. **Results**: Notably, positive marital interaction buffered the association between perceived stress and depressive symptoms, whereas marital disagreement exacerbated this association. **Conclusions**: These findings underscore the dual role of marital quality in buffering or exacerbating the association between perceived stress and depressive symptoms, highlighting the importance of promoting marital quality as a potential approach to improving the psychological well-being of urban Chinese women.

## 1. Introduction

Since the 2010s, the prevalence of depressive symptoms in China has risen markedly [1]. This increase is particularly pronounced among women [2,3], which has been partly attributed to the cumulative pressures stemming from both professional and domestic responsibilities [4,5,6]. Depression represents a major source of disease-related disability worldwide, with women experiencing a consistently greater prevalence and burden than men [7,8]. Its consequences can extend beyond individual well-being, as maternal depression is associated with adverse emotional and developmental outcomes among children [9,10,11]. Accordingly, addressing depressive symptoms among women and promoting their mental health has become an important area of research. This study examines the moderating role of marital quality, an integral facet of marital well-being, in the association between perceived stress and depressive symptoms among married women in urban China, aiming to advance understanding of the relationship between mental health and marital quality in contemporary Chinese society.

Stressful events are common, yet individuals differ in their cognitive appraisals of external stressors. Perceived stress, defined as “the degree to which situations in one’s life are appraised as stressful” [12], has been widely recognized as a key predictor of depressive symptoms [13,14,15,16,17,18]. Notably, perceived stress demonstrates pronounced gender disparities, with women reporting consistently higher levels than men [19,20]. This heightened perceived stress among women is associated with increased severity of depressive symptoms [21,22].

Previous research has highlighted the role of marriage and family as important social contexts influencing depressive symptoms and their gender differences. Existing studies show that both marriage and cohabitation with a partner are associated with a lower risk of depressive symptoms for both genders [23,24]. However, research findings reveal that marital status alone does not uniformly confer mental health advantages; rather, the quality of the marital relationship is a more salient determinant [25,26,27]. In effect, high-quality marital relationships are associated with superior mental health outcomes compared to remaining single, with pronounced benefits observed among women [28].

While prior research has investigated the association between perceived stress and depressive symptoms, the role of marital quality as a moderating factor remains understudied. This gap is particularly relevant in Chinese sociocultural contexts, where marriage has undergone substantial changes from a traditionally family-oriented institution toward a more individualized conjugal relationship. Consequently, the moderating influence of marital quality on depressive symptoms among women warrants further scholarly attention. This study thus examines the complex interrelations among perceived stress, marital quality, and depressive symptoms in married women residing in urban China. We argue that the positive dimension of marital quality, namely positive marital interaction, serves as a vital social and personal resource to help cope with or buffer the deleterious effects of perceived stress on mental health. Conversely, the negative dimension of marital quality, namely marital disagreement, may exacerbate these detrimental effects. Therefore, marriage is capable of both enhancing and impairing mental health outcomes. By distinguishing positive and negative dimensions of marital quality, this study contributes to a better understanding of how intimate relationships may buffer or amplify the association between perceived stress and depressive symptoms among urban married women in China.

## 2. Theoretical Framework

### 2.1. Marital Quality and Depressive Symptoms

Over the past several decades, marriage and family in China have undergone profound structural and relational changes. Ji [29] characterizes contemporary Chinese familialism as a multifaceted construct shaped by enduring patriarchal traditions, socialist legacies, market-driven dynamics, and neoliberalism. Structurally, there has been a marked decline in traditional extended and multigenerational family forms, accompanied by a rise in nuclear family arrangements [30]. Spousal relationships have increasingly supplanted the conventional parent–child nexus as the focal point of family life [31]. In terms of family values and relationships, the conceptualization of family in China has shifted from a “quasi-religion” institution grounded in filial piety [32] to one oriented toward individualized emotional fulfillment. Prior to China’s Reform and Opening-Up, prevailing social norms strongly stigmatized divorce, resulting in the persistence of many marriages despite suboptimal quality. During this period, marital quality was predominantly assessed through stability indicators such as divorce rates. In the post-reform era, driven by neoliberal transformations and the inherent tensions of traditional familialism, China is witnessing a deinstitutionalization of marriage that parallels Western patterns, including declining marriage rates, increasing divorce incidence, and growing cohabitational unions [33,34,35]. Against this backdrop, younger generations have begun to prioritize emotional experiences over familial obligations within marriage, thereby elevating expectations for marital quality. For urban married women, who negotiate the complex interplay between traditional obligations and modern autonomy, these individualized and privatized marital relationships constitute key sources of either social support or social undermining (i.e., detriment).

Marital quality refers to an individual’s subjective and evaluative perceptions of their marriage or partnership [36]. Traditionally, marital quality has been assessed using unidimensional indicators such as “marital happiness” or “marital satisfaction,” which inadequately capture the multifaceted nature of relational dynamics. A more comprehensive bidimensional framework, encompassing both positive and negative assessments, provides a more nuanced conceptualization of marital quality [37,38,39]. The positive dimension includes such aspects as happiness, satisfaction, intimacy, consensus, agreement, autonomy, harmony, and sexual relationships, whereas the negative dimension pertains to conflict, problems, or disagreements [40]. These dimensions are conceptualized as distinct constructs, exhibiting, at most, a moderate inverse correlation [37].

Extensive research has demonstrated significant associations between marital quality and depressive symptoms, as well as broader psychological well-being [25,26]. From the standpoint of marital quality, marriage does not necessarily confer mental health benefits. Stressors are perceived across multiple levels, namely micro, meso, and macro [41], with both discrete events and ongoing life circumstances contributing to stress [42]. At the micro level, even everyday trivialities may act as chronic stressors. Marital and family conflicts, for example, represent enduring or recurrent stressors frequently encountered in everyday life [43]. Consequently, ostensibly trivial aspects of marital interactions can precipitate episodes of depressive symptoms [44]. Individuals in harmonious marital relationships tend to exhibit fewer health complications [45], whereas those experiencing relationship discord are more susceptible to psychological difficulties [46,47].

### 2.2. The Stress Process Model

The stress process model is a seminal theoretical framework that has profoundly influenced sociological research on stress and mental health [48,49]. Pearlin et al. [42] pioneered the conceptualization of the “stress process,” integrating diverse components of social stress affecting individuals into a unified theoretical model encompassing three primary domains: sources of stress, mediators and moderators of stress, and manifestations of stress. Pearlin posits that the social determinants and patterns underlying health and illness emerge from the dynamic interplay between stressors (or strains) and available resources.

In the epidemiologic tradition, research on stress has predominantly focused on relatively objective stressors arising from individual life events [50]. Conversely, the psychological tradition emphasizes the dynamic interplay between individuals and their environment, wherein individuals appraise potential threats based on their available coping resources [50,51,52]. Stress is thus conceptualized not as an inherent property of the environment but as the result of a discrepancy between environmental demands and an individual’s capacity to adapt [53]. Consequently, the impact of stressors manifests only when situations are appraised as threatening and coping resources are insufficient to manage these demands [12,50]. The Perceived Stress Scale (PSS) is widely used to assess the subjective experience of stress [12,54] and has demonstrated reliability and predictive validity for morbidity and mortality outcomes [55,56,57,58].

Drawing on the stress process model, we propose the first conceptual model, referred to as the “main effect model,” which posits a positive association between perceived stress and depressive symptoms among married women in urban China. Accordingly, we propose the following hypothesis:

**Hypothesis 1.** 
*Higher levels of perceived stress will be positively associated with increased depressive symptoms among married women in urban China (main effect model)*.

Mediators and moderators in the stress process model include social resources, personal resources, and coping strategies [26]. However, these resources do not necessarily provide support to individuals; their effectiveness depends not only on the extent of social network integration but also on the quality of relationships within those networks. This framework shifts the focus from individual deficits to the broader, stress-inducing environmental context. Within marital relationships, a high-quality marriage is consistently associated with better well-being [59] through mutual economic and social support that helps buffer perceived stress [45]. Conversely, marital instability may increase psychological distress and contribute to more severe psychiatric disorders [60,61]. Building on the stress process model, scholars have further proposed the stress-buffering and stress-exacerbating hypotheses to examine the moderating role of social resources [62,63,64,65], which are reviewed below.

#### 2.2.1. The Stress-Buffering Hypothesis

The stress-buffering hypothesis, as a fundamental element of the stress process model, posits that specific resources can shield individuals from the adverse effects of stressors or strains [65]. These resources serve as a protective buffer between stressors and mental health outcomes. Furthermore, perceived social support, as opposed to received social support, plays a more direct and significant role in mitigating the detrimental impact of major life events and chronic stressors on mental health [62,66,67]. In this context, marital support, particularly emotional support characterized by shared time, emotional exchange, mutual care, and fulfillment of needs, helps attenuate the relationship between stressors and depressive symptoms [68,69].

#### 2.2.2. Stress-Exacerbating Hypothesis

The stress-exacerbation hypothesis builds on the stress-buffering hypothesis by positing that the deleterious impact of stress on depressive symptoms is intensified among individuals experiencing elevated levels of social strain [70]. In the context of marriage, spouse support and spouse undermining represent distinct conceptual constructs, each exerting differential effects on depressive symptoms. Longitudinal research by Cranford [64] indicates that spouse undermining, rather than spouse support, significantly exacerbated the effects of stressors on depressive symptoms. Marital relationships characterized by high conflict can generate chronic stress, which can deteriorate support networks and compromise individuals’ coping capacities, thereby amplifying emotional distress [71]. These dynamics may further increase psychological distress, contributing to the heightened severity of depressive symptoms [72]. Moreover, gender role expectations render women more sensitive to stress, and consequently, more susceptible to the adverse mental health consequences of dysfunctional marital relationships [73]. While the buffering effects of social support have been extensively studied, the exacerbating effects have garnered comparatively less empirical scrutiny [74]. However, studies specifically addressing the stress-exacerbation hypothesis among Chinese populations remain scarce.

**Figure 1:**
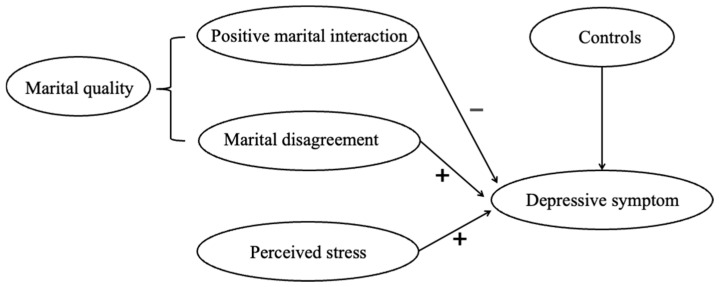
Offsetting/enhancing effect model.

**Figure 2:**
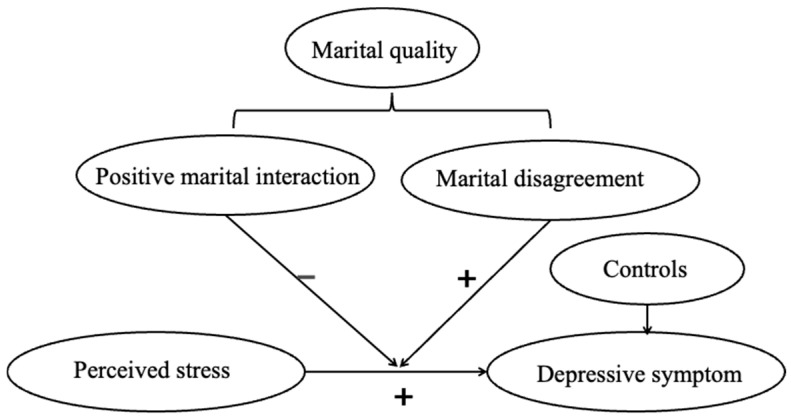
Stress-buffering/exacerbating model.

The literature reviewed above indicates that marital quality serves as a moderator in the relationship between perceived stress and depressive symptoms. To further examine this moderating role among urban marriages in China, we propose two additional conceptual models. The second conceptual model, termed the “offsetting/enhancing effect model,” examines whether positive marital quality offsets whereas negative marital quality strengthens the association of perceived stress with depressive symptoms, as shown in Figure 1. The third conceptual model, referred to as the “stress-buffering/exacerbating model,” assesses the interaction effects between perceived stress and both positive and negative dimensions of marital quality. This model underscores the moderating role of marital quality in the association between perceived stress and depressive symptoms, as depicted in Figure 2. Guided by these conceptual models, we propose and test the following two hypotheses:

**Hypothesis 2.** 
*Marital quality will offset or enhance the effects of perceived stress on depressive symptoms (offsetting/enhancing effect model)*.

**Hypothesis 3.** 
*The association between perceived stress and depressive symptoms will be moderated by marital quality, such that marital quality either buffers or exacerbates this association (stress-buffering/exacerbating model)*.

## 3. Materials and Methods

### 3.1. Data and Sample

This study used data from the 2020 Family and Health Survey conducted in Chengdu and Beijing, China. This survey served as a follow-up and update to the 1987 Family Life Survey conducted in Chengdu by the University of Michigan [75]. The 1987 Chengdu survey itself was designed to replicate the 1984 Detroit area study, also conducted by the University of Michigan [76]. The 2020 survey was collaboratively conducted by research teams from multiple universities in China and the United States. Chengdu was selected as a survey site to facilitate a 33-year longitudinal follow-up of the 1987 Chengdu study, while Beijing was included due to its political and economic prominence as the capital of China, allowing for comparative analyses with the Chengdu data. The survey protocol received approval from a university-affiliated hospital ethics committee in China (Approval No. K2019032). All participants provided informed consent prior to participation and received modest compensation.

The study used a multistage random sampling methodology. In Chengdu and Beijing, residential communities were randomly selected, and households were then sampled using systematic procedures. From each household, one married woman who had lived in the city for at least six months was selected. A total of 2086 ever-married women aged 20–99 were interviewed, 821 from Chengdu and 1265 from Beijing. Trained interviewers conducted face-to-face surveys, and all respondents completed the questionnaires independently without interference from other household members. The sampling process and data collection were conducted by a professional survey and research consulting firm, Chengdu Sunbird Survey & Research Co., Ltd. (Chengdu, China). Owing to the face-to-face design, no item-level missing data were observed for the PHQ-9, PSS-10, or marital quality measures. However, because the analysis focused on current marital quality, 221 respondents who were divorced or widowed were excluded, resulting in a final sample of 1865 currently married women.

Table 1 presents the demographic and psychosocial characteristics of the final analytic sample from Chengdu and Beijing. The mean age of participants at the time of the interview was 45.6 years, with an average educational attainment of 12 years. Respondents reported having slightly more than one child on average, and approximately 63% were employed. The majority of respondents (73.73%) reported no or minimal depressive symptoms. Additionally, the mean perceived stress score was 15.1, indicative of a moderate stress level [54]. Regarding marital quality, the mean score on the positive marital interaction index was 1.2, suggesting that respondents engaged with their spouses sometimes or often. In contrast, the mean score on the marital disagreement index was 0.5, indicating that respondents experienced marital disagreements infrequently or not at all. Compared with the 221 respondents excluded from the analysis (Supplementary Table S1), the analytical sample had lower mean depressive symptoms (PHQ-9: 3.36 vs. 4.28) and perceived stress (PSS-10: 15.09 vs. 15.54), higher positive marital interaction (1.17 vs. 0.76), and lower marital disagreement (0.52 vs. 0.59).

### 3.2. Measures

DV: depressive symptoms. Depressive symptoms were measured using the nine-item Patient Health Questionnaire (PHQ-9). Each item was rated based on symptom frequency over the preceding two weeks, using a four-point Likert scale ranging from 0 (not at all) to 3 (nearly every day). The composite score, derived by summing the nine items, served as an index of depressive symptom severity, with higher scores indicating greater severity. The PHQ-9 total score is conventionally categorized into severity levels as follows: 0–4 (minimal), 5–9 (mild), 10–14 (moderate), 15–19 (moderately severe), and 20 or greater (severe) [77]. For this study, the continuous PHQ-9 score was used instead of categorical groupings. The Cronbach’s alpha coefficient for the PHQ-9 was 0.875.

IV: perceived stress. Perceived stress was measured using the 10-item Perceived Stress Scale (PSS-10) [12,54], which demonstrates superior psychometric properties compared to the 14-item version [54,78]. The PSS-10 captures dimensions of unpredictability, uncontrollability, and overload in individuals’ personal lives. Participants rated their perceived stress over the preceding month using a five-point Likert scale ranging from 0 (never) to 4 (very often). Four positively worded items in the PSS-10 scale were reverse-coded. The total perceived stress score was calculated by summing responses across all ten items, with higher scores indicating greater perceived stress levels. The Cronbach’s alpha coefficient for the PSS-10 was 0.872.

Moderating variables: Marital quality was measured using two distinct dimensions: positive marital interaction and marital disagreement [37,38,39]. These dimensions were derived from the multidimensional conceptualization of marital quality proposed by Johnson et al. [38] and were subsequently incorporated into the Chinese Marital Quality Scale (CMQS), which was developed and validated in Chinese populations by Xu [79] and Zhang et al. [39]. Because the present study used a later wave of the same Family and Health Survey from which the CMQS was originally developed, we adopted these two dimensions with only minor modifications based on data availability. Positive marital interaction was measured using six items: (1) frequency of spending time with one’s spouse outside of work-related contexts; (2) frequency with which the spouse shares personal thoughts and feelings; (3) perceived feelings of being loved by the spouse; (4) satisfaction with the sexual relationship; (5) spouse’s attentiveness to the participant’s problems and concerns; and (6) frequency of discussions about marital issues. Participants rated each item on a three-point scale (1 = “Very often,” 2 = “Sometimes,” 3 = “Rarely”), with responses reverse-coded (0 = “Rarely,” 1 = “Sometimes,” 2 = “Very often”) so that higher scores indicated more positive marital interactions. Marital disagreement was assessed via seven items addressing: (1) division of household labor; (2) decision regarding childbearing; (3) disciplinary approaches for children; (4) care for elderly parents; (5) management of interactions with members of the opposite sex; (6) frequency of arguments about sexual issues; and (7) disputes concerning property or housing ownership. These items were rated on a three-point scale (1 = “Often,” 2 = “Sometimes,” 3 = “Never”), with reverse coding applied (0 = “Never,” 1 = “Sometimes,” 2 = “Often”) such that higher scores reflected greater marital disagreement. Both scales demonstrated high internal consistency reliability, with Cronbach’s alpha coefficients of 0.835 for positive marital interaction and 0.866 for marital disagreement. Confirmatory factor analysis (CFA) using structural equation modeling (SEM) further supported the construct validity of the two marital quality scales. All standardized factor loadings exceeded 0.50, ranging from 0.56 to 0.77 for positive marital interaction and from 0.53 to 0.76 for marital disagreement.

Sociodemographic controls: This study includes several sociodemographic variables as statistical controls: age, educational attainment, employment status (coded as 1 for employed and 0 for unemployed), log-transformed couple’s monthly income, household size, number of children, self-assessed health status, self-rated social class standing (measured on a scale from 1 to 10), and survey location (coded as 1 for Beijing and 0 for Chengdu).

### 3.3. Data Analysis

Using the Ordinary Least Squares (OLS) regression technique, we estimated five nested regression models. This approach was selected because the primary objective was to examine the incremental contributions of distinct dimensions of marital quality and their moderating effects on the association between perceived stress and depressive symptoms. Positive marital interaction and marital disagreement were analyzed separately because they are conceptualized as distinct dimensions of marital quality rather than opposite ends of a single continuum. Model 1 (main effect model) tests Hypothesis 1 by examining the direct regression relationship between perceived stress and depressive symptoms, controlling for relevant covariates. Models 2 and 3 (offsetting/enhancing effect models) incorporate positive marital interaction and marital disagreement, respectively, to evaluate the hypothesized offsetting or enhancing effects. Lastly, Models 4 and 5 (buffering/exacerbating models) introduce interaction terms between perceived stress and both positive marital interaction and marital disagreement to investigate potential stress buffering or exacerbating effects. Continuous predictors were mean-centered prior to analysis, while PHQ-9 remained on its original scale. Although PHQ-9 scores are right-skewed, they represent a bounded psychometric scale measuring depressive symptom severity rather than event counts. Accordingly, OLS regression with robust standard errors was used to estimate associations with the continuous PHQ-9 score. As a robustness check, all models were re-estimated using a log-transformed PHQ-9 score, yielding substantively similar results (Supplementary Table S2). All statistical analyses were conducted using Stata 16 (StataCorp LLC, College Station, TX, USA).

## 4. Results

Model 1 included in Table 2 illustrates the findings from the main effect model. Consistent with the hypothesis, after controlling for sociodemographic variables, perceived stress was significantly and positively associated with depressive symptoms. Specifically, all else being equal, a one-point increase in perceived stress corresponds to a 0.442-point estimated increase in PHQ-9 score (*p* < 0.001). This result is consistent with Hypothesis 1.

Model 2 in Table 2 incorporates positive marital interaction into Model 1 to examine its potential offsetting effect. The result indicates that increased levels of positive marital interaction were significantly associated with reduced depressive symptoms. That is, for each one-point increase in positive marital interaction, the estimated PHQ-9 score decreased by 0.461 units (*p* < 0.01), after adjusting for all sociodemographic variables. The finding suggests that positive marital interaction was associated with lower levels of depressive symptoms beyond the effect of perceived stress. Consequently, the findings were consistent with the offsetting hypothesis (Hypothesis 2).

Model 3 presented in Table 2 adds marital disagreement to Model 1 to evaluate the enhancing effect. Consistent with the hypothesis, increased levels of marital disagreement were significantly associated with elevated depressive symptoms. Specifically, for each one-unit increment in marital disagreement, the estimated depressive symptoms (PHQ-9) increased by 0.777 units, controlling for sociodemographic characteristics (*p* < 0.001). These results were consistent with the enhancing effect hypothesis (Hypothesis 2).

**Figure 3:**
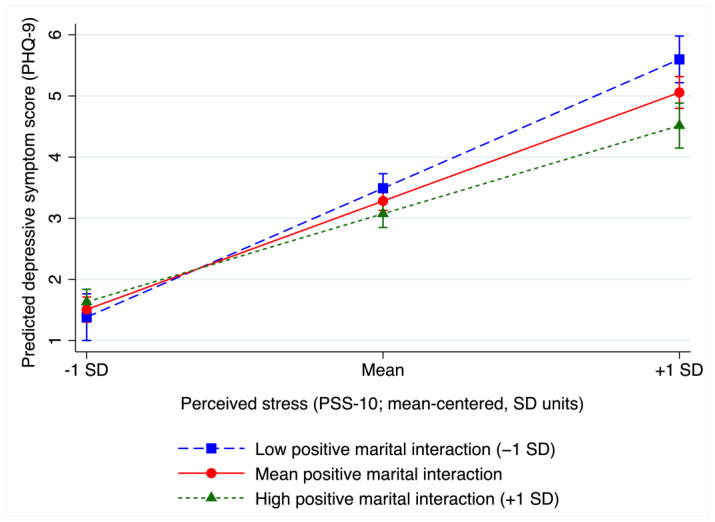
Association between perceived stress and predicted depressive symptoms by levels of positive marital interaction. Lines represent predicted PHQ-9 scores at one standard deviation below the mean (−1 SD), at the mean, and one standard deviation above the mean (+1 SD) of positive marital interaction.

Model 4, as shown in Table 2, adds the interaction term between perceived stress and positive marital interaction to Model 2 to test the buffering hypothesis. The interaction term was statistically significant (B = −0.165, *p* < 0.001), indicating that positive marital interaction moderated the association between perceived stress and depressive symptoms. Specifically, as the level of positive interaction increased, the association between perceived stress and depressive symptoms became weaker. This moderating effect is further elucidated in Figure 3, which visually depicts how positive marital interaction attenuated the association between perceived stress and depressive symptoms. Consequently, these findings were consistent with the buffering hypothesis (Hypothesis 3).

**Figure 4:**
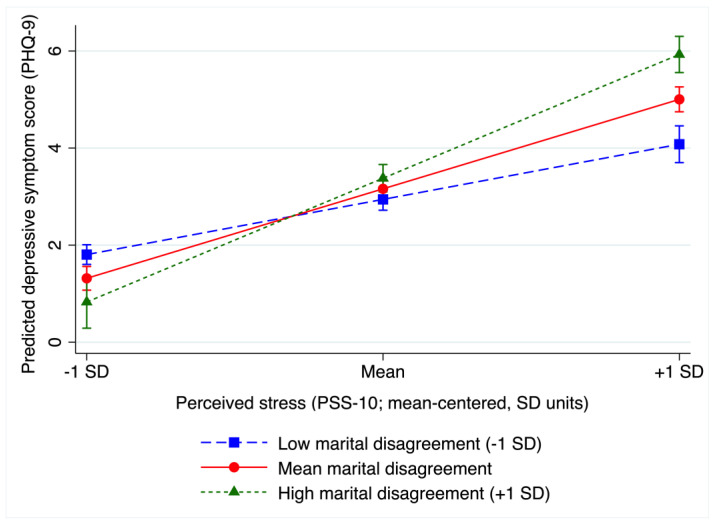
Associations between perceived stress and predicted depressive symptoms by levels of marital disagreement. Lines represent predicted PHQ-9 scores at one standard deviation below the mean (−1 SD), at the mean, and one standard deviation above the mean (+1 SD) of marital disagreement.

Model 5, presented in Table 2, incorporates an interaction term between perceived stress and marital disagreement to test the exacerbating hypothesis. Consistent with expectations, this interaction term was statistically significant (B = 0.373; *p* < 0.001), indicating that marital disagreement strengthened the association between perceived stress and depressive symptoms. That is, as marital disagreement increased, the association between perceived stress and depressive symptoms became stronger. This interaction pattern is visually depicted in Figure 4. Collectively, these findings are consistent with Hypothesis 3.

## 5. Discussion

This study investigated three conceptual models examining the relationships among perceived stress, marital resources (i.e., marital support and marital undermining), and depressive symptoms among married women in urban China. Guided by the stress process model, the first conceptual model, the “main effect model,” examined the association between perceived stress (PSS) and depressive symptoms (PHQ-9) while controlling for sociodemographic characteristics. Regression analyses using survey data collected from two major Chinese metropolitan areas, Chengdu and Beijing, demonstrated a significant positive association between perceived stress and depressive symptoms. This finding is consistent with previous research documenting the association between perceived stress and adverse mental health outcomes, including depressive symptoms, in both the general Chinese population and industrialized European contexts [16]. Importantly, our results highlight the detrimental mental health implications for married Chinese women who navigate multiple concurrent stressors related to occupational demands, parenting responsibilities, and domestic or familial obligations on a daily basis.

The second conceptual model, termed the “offsetting or enhancing model,” extended beyond the main effect model by examining the potential roles of positive and negative dimensions of marital quality. We proposed that positive marital quality, reflected by positive marital interactions as an indicator of marital support, would exhibit a negative association with depressive symptoms, whereas negative marital quality, reflected by marital disagreement as an indicator of marital undermining, would be positively associated with depressive symptoms. Our analyses provided support for these hypotheses among married women in urban China. These findings suggest that marital support, encompassing elements such as companionship, shared experiences and feelings, and mutual care, is associated with lower levels of depressive symptoms, whereas frequent marital disagreement is associated with higher levels of depressive symptoms among married women in China.

The third conceptual model was the “buffering or exacerbation model.” This model posited that the association between perceived stress and depressive symptoms was weaker for married women who experienced higher marital quality, reflecting greater relational resources. Conversely, the association between perceived stress and depressive symptoms was stronger for married women characterized by lower marital quality, reflected by greater marital disagreement and fewer relational resources. Accordingly, we hypothesized that marital quality, as a distinct form of social support and resource, could be associated with either attenuating or amplifying the association between perceived stress and the mental health of married Chinese women. Our regression analyses supported both the buffering and exacerbation hypotheses. Specifically, in urban Chinese marriages where wives reported higher levels of positive marital quality, the association between perceived stress and depressive symptoms was significantly weaker. In contrast, in urban Chinese marriages where wives reported higher levels of marital disagreement, the association between perceived stress and depressive symptoms was significantly stronger.

What mechanisms, then, help explain the capacity of marriages characterized by high positive interactions to buffer the adverse association between perceived stress and depressive symptoms within Chinese marital contexts? In contemporary China, particularly in urban settings, marital unions have evolved from traditional family-centered frameworks toward individualized, privatized, and emotionally nuanced partnerships in the post-reform era. Consequently, positive marital interactions in China may be understood similarly to those observed in American or Western contexts. Specifically, such interactions provide important social, marital, and emotional support that helps reduce the detrimental impacts of stressful life events and conditions [80,81]. Furthermore, positive marital interactions reflect strong emotional bonds and effective marital communication. These dynamics enable spouses to develop psychological resources, coping skills, and a stronger sense of control and mastery over life circumstances [82,83,84]. Marriages marked by frequent positive interactions and minimal conflict can influence spouses’ behaviors and lifestyles, thereby reducing their vulnerability to specific social stressors. For example, partners may mutually regulate behaviors to avoid risk-taking and unhealthy habits, including substance abuse and poor dietary choices [85,86,87,88,89]. Additionally, spouses can encourage each other to seek help, which may serve as a protective factor against severe mental health issues.

Although marital quality significantly moderated the association between perceived stress and depressive symptoms in this study, the moderating effects were smaller than the direct effects, suggesting that marriage represents only one component of women’s broader social support system. Consistent with the relational structure of Chinese society, support is often embedded in multiple social ties beyond the marital relationship, including kinship and friendship networks [90,91], both of which have been linked to better mental health among Chinese adults [92,93]. The remaining unexplained variance may partly reflect interpersonal resources beyond marital quality, as well as other unmeasured social, psychological, and health-related factors.

These findings have several theoretical implications. They extend the application of the stress process perspective to urban Chinese marriages. First, marital quality plays multiple roles within the stress process, functioning both as an independent stressor and a contextual factor shaping responses to perceived stress. Second, the findings support treating positive and negative dimensions of marital quality as distinct relational processes. The quality of marital relationships can provide substantial mental health benefits, yet it also has the potential to undermine the psychological well-being of married women. Marriages characterized by instability, frequent disagreements, or chronic conflict may contribute to maladaptive coping behaviors [64]. Consequently, low-quality marital relationships can become significant sources of stress, introducing stressors that may exacerbate negative affect and elevate the risk of depressive symptoms.

The findings have several practical implications for public mental health policies and family services in urban China. Mental health interventions targeting married women may benefit from extending beyond individual stress management to include couple-based approaches that promote positive communication and constructive conflict resolution. Integrating these relational components into community mental health services or marital counseling programs may help strengthen women’s psychological resilience to stress and reduce depressive symptoms.

Several limitations of this study warrant consideration. First, the use of cross-sectional survey data constrains the ability to draw causal inferences. Accordingly, the observed moderating relationships should be interpreted as associational rather than causal. Prior research indicates that the association between marital quality and depressive symptoms may be bidirectional, such that elevated depressive symptoms can also diminish marital satisfaction [94,95]. Consequently, clarifying the causal direction between these constructs requires more rigorous investigation through longitudinal research designs. Second, the sample consisted exclusively of married women residing in urban China. This limitation prevents assessment of whether the observed associations among perceived stress, marital quality, and depressive symptoms operate similarly for married men in comparable contexts. Future studies should incorporate data from both spouses to evaluate these dynamics within a dyadic framework. Third, the data were drawn from two major cities representing Southwestern and Northern China. Given substantial regional heterogeneity, the generalizability of the findings to rural populations and other regions of China should be interpreted with caution. Nationally representative samples would strengthen the external validity of future research. Fourth, all key variables were measured through self-reported questionnaires, which may have introduced common method variance and reporting bias. Future studies could strengthen the evidence by incorporating multiple data sources or partner-reported assessments of marital quality. Finally, to more fully evaluate the buffering and exacerbation hypotheses regarding perceived stress and depressive symptoms, future studies should examine additional sources of social support, including friendship and kinship networks, participation in neighborhood or community activities, and involvement in broader social or professional associations.

## 6. Conclusions

This study advances cross-cultural research on perceived stress, marital dynamics, and depressive symptoms by examining how marital quality, measured by positive interactions and marital disagreement, moderates the association between perceived stress and depressive symptoms among married women in urban China. By examining these relationships in the context of rapid social change in urban China, this study suggests that the stress-buffering/exacerbating function of marriage should be understood as relational rather than structural. The findings highlight the important role of social and emotional resources embedded within marital relationships in women’s responses to stress-related depressive symptoms. Overall, these findings provide a foundation for developing relationship-oriented strategies to strengthen women’s resilience to stress and promote mental health among married women.

## Figures and Tables

**Table 1:** Sample characteristics (N = 1865).

	N	%	Mean	SD	Min	Max
PHQ-9	1865		3.36	3.84	0	23
0–4 (minimal)	1375	73.73				
5–9 (mild)	341	18.28				
10–14 (moderate)	110	5.90				
15–19 (moderately severe)	37	1.98				
20 or greater (severe)	2	0.11				
Perceived stress scale (PSS-10)	1865		15.09	3.99	0	27
Positive marital interaction (Index)	1865		1.17	0.51	0	2
Marital disagreement (Index)	1865		0.52	0.47	0	2
Age	1865		45.62	13.73	20	99
Years of education	1865		12.01	3.92	0	19
Couple monthly income (log)	1865		9.07	0.78	5.30	11.51
Number of children	1865		1.06	0.75	0	4
Number of household members	1865		2.72	1.13	1	6
Self-identified class	1865		4.56	1.96	1	10
Employment status						
Employed	1177	63.11				
Not working	688	36.89				
Survey location						
Chengdu	751	40.27				
Beijing	1114	59.73				

**Table 2:** OLS regression models to predict depressive symptoms (robust standard errors).

	Main Effects Model	Offsetting Effects Model	Enhancing Effects Model	Buffering Effect Model	Exacerbating Effect Model
	Model 1	Model 2	Model 3	Model 4	Model 5
Perceived Stress Scale (PSS)	0.442 ***	0.431 ***	0.418 ***	0.444 ***	0.462 ***
	(0.021)	(0.021)	(0.022)	(0.023)	(0.024)
Positive marital interaction		−0.461 **		−0.412 *	
		(0.178)		(0.676)	
Marital disagreement			0.777 ***		0.460 *
			(0.209)		(0.212)
PSS × Positive Interaction				−0.165 ***	
				(0.045)	
PSS × Disagreement					0.373 ***
					(0.061)
Age	−0.001	−0.002	−0.001	−0.002	−0.001
	(0.009)	(0.009)	(0.009)	(0.009)	(0.009)
Educational attainment	−0.052	−0.047	−0.043	−0.051	−0.054
	(0.029)	(0.028)	(0.028)	(0.028)	(0.028)
Currently employed (=1) (Ref: Unemployed = 0)	0.108	0.094	0.067	0.094	0.113
	(0.210)	(0.211)	(0.209)	(0.210)	(0.206)
Couple monthly income (log)	−0.077	−0.055	−0.031	−0.082	−0.034
	(0.143)	(0.143)	(0.144)	(0.143)	(0.140)
Num. of household member	−0.363 ***	−0.362 ***	−0.330 ***	−0.344 ***	−0.306 ***
	(0.075)	(0.075)	(0.074)	(0.075)	(0.074)
Num. of children	−0.055	−0.078	−0.036	−0.084	0.013
	(0.140)	(0.141)	(0.140)	(0.140)	(0.138)
Self-rated health	−0.409 ***	−0.369 ***	−0.453 ***	−0.388 ***	−0.504 ***
	(0.104)	(0.105)	(0.103)	(0.104)	(0.101)
Self-rated Social Class Standing	0.067	0.074	0.054	0.082	0.072
	(0.051)	(0.051)	(0.051)	(0.051)	(0.050)
Beijing (=1) (Ref: Chengdu = 0)	0.580 ***	0.620 ***	0.476 **	0.615 ***	0.390 **
	(0.154)	(0.155)	(0.151)	(0.155)	(0.151)
Constant	7.167 ***	6.754 ***	6.931 ***	6.956 ***	6.958 ***
	(1.443)	(1.445)	(1.436)	(1.434)	(1.407)
F	59.48 ***	54.11 ***	57.88 ***	55.28 ***	59.03 ***
R^2^	0.269	0.272	0.277	0.279	0.296
N	1865	1865	1865	1865	1865

Note: * *p* < 0.05. ** *p* < 0.01. *** *p* < 0.001. Robust SE statistics in parentheses. PSS: Perceived Stress Scale. Across all five models, the maximum VIF was 2.07 (mean VIF = 1.36), indicating no evidence of problematic multicollinearity.

## Data Availability

The data analyzed in this study are not publicly available due to data use agreements and ethical/privacy restrictions. Researchers may obtain access to the data from the corresponding data providers upon reasonable request and approval.

## Supplementary Materials

The following supporting information can be downloaded at: https://www.mdpi.com/article/10.3390/healthcare14152246/s1, Table S1: Characteristics of respondents excluded from the analysis (N = 221); Table S2: Robustness check: OLS regression models using the log-transformed PHQ-9 score.
