# Supplementary material for: Perceived Stress and Depressive Symptoms in Urban Chinese Women: The Buffering and Exacerbating Roles of Marital Quality
Source: Healthcare (Basel). 2026 Jul 23;14(15):2246. doi: 10.3390/healthcare14152246 (PMC13463907; doi:10.3390/healthcare14152246)
Supplement: Supplementary file 1 [file healthcare-14-02246-s001.zip › healthcare-4372134-supplementary.pdf]

Supplementary

Table S1. Characteristics of respondents excluded from the analysis (N = 221).

	N	%	Mean	SD	Min	Max
PHQ-9	221		4.28	3.38	0	17
0–4 (minimal)						
5–9 (mild)						
10–14 (moderate)						
15–19 (moderately severe)						
20 or greater (severe)						
Perceived stress scale (PSS-10)	221		15.54	3.92	2	25
Positive marital interaction (Index)	221		0.76	0.48	0	2
Marital disagreement (Index)	221		0.59	0.39	0	2
Age	221		54.31	12.85	23	82
Years of education	221		10.30	4.47	0	19
Couple monthly income (log)	221		8.24	1.00	5.30	11.92
Number of children	221		1.30	1.09	0	6
Number of household members	221		1.89	1.14	1	6
Self-identified class	221		3.57	1.72	1	8
Employment status						
Employed	100	45.25				
Not working	121	54.75				
Survey location						
Chengdu	70	31.67				
Beijing	151	68.33				

Table S2. Robustness check: OLS regression models using the log-transformed PHQ-9 score.

	Main Effects Model	Offsetting Effects Models	Enhancing Effects Models	Buffering Effect Models	Exacerbating Effect Models
	Model 1	Model 2	Model 3	Model 4	Model 5
Perceived Stress Scale (PSS)	0.032***	0.031***	0.030***	0.032***	0.033***
	(0.001)	(0.001)	(0.001)	(0.002)	(0.002)
Positive marital interaction		-0.034**		-0.031*	
		(0.013)		(0.013)	
Marital disagreement			0.058***		0.038*
			(0.015)		(0.015)
PSS × Positive Interaction				-0.009**	
				(0.003)	
PSS × Disagreement					0.023***
					(0.004)
Age	0.000	-0.000	0.000	-0.000	0.000
	(0.001)	(0.001)	(0.001)	(0.001)	(0.001)
Educational attainment	-0.003	-0.002	-0.002	-0.003	-0.003
	(0.002)	(0.002)	(0.002)	(0.002)	(0.002)
Currently employed (=1)	0.007	0.006	0.004	0.006	0.007
(Ref: Unemployed =0)	(0.015)	(0.015)	(0.014)	(0.015)	(0.014)
Couple monthly income (log)	-0.077	-0.055	-0.031	-0.082	-0.034
	(0.143)	(0.143)	(0.144)	(0.143)	(0.140)
Num. of household member	-0.021***	-0.021***	-0.019***	-0.020***	-0.017***
	(0.005)	(0.005)	(0.005)	(0.005)	(0.005)
Num. of children	0.003	0.001	0.004	0.001	0.007
	(0.010)	(0.010)	(0.010)	(0.010)	(0.009)
Self-rated health	-0.033***	-0.030***	-0.037***	-0.031***	-0.040***
	(0.007)	(0.007)	(0.007)	(0.007)	(0.007)
Self-rated Social Class Standing	0.005	0.006	0.004	0.006	0.005
	(0.003)	(0.003)	(0.003)	(0.003)	(0.003)
Beijing(=1)	0.024*	0.027*	0.017	0.027*	0.011
(Ref: Chengdu =0)	(0.011)	(0.011)	(0.011)	(0.011)	(0.011)
Constant	2.740***	2.709***	2.722***	2.720***	2.724***
	(0.097)	(0.097)	(0.096)	(0.097)	(0.095)
F	74.2***	67.91***	71.35***	66.59***	71.40***
R^2	0.276	0.280	0.285	0.284	0.300
N	1,865	1,865	1,865	1,865	1,865

Note: *p < .05. **p < .01. ***p < .001. Robust SE statistics in parentheses. PSS: Perceived Stress Scale. As a robustness check, the dependent variable was replaced with a log-transformed PHQ-9 score. PHQ-9 items were recoded from 0–3 to 1–4 before summation and natural log transformation. All other model specifications remained identical to those in the main analyses.
